# Translational implications of *CHRFAM7A*, an elusive human-restricted fusion gene

**DOI:** 10.1038/s41380-023-02389-1

**Published:** 2024-01-10

**Authors:** Ivanna Ihnatovych, Ruth-Ann Saddler, Norbert Sule, Kinga Szigeti

**Affiliations:** 1 Department of Neurology, State University of New York at Buffalo, 875 Ellicott St., Buffalo, NY 14203 USA; 2 Roswell Park Comprehensive Cancer Center, 665 Elm St, Buffalo, NY 14203 USA

**Keywords:** Neuroscience, Genetics, Diseases

## Abstract

Genes restricted to humans may contribute to human-specific traits and provide a different context for diseases. *CHRFAM7A* is a uniquely human fusion gene and a negative regulator of the α7 nicotinic acetylcholine receptor (α7 nAChR). The α7 nAChR has been a promising target for diseases affecting cognition and higher cortical functions, however, the treatment effect observed in animal models failed to translate into human clinical trials. As *CHRFAM7A* was not accounted for in preclinical drug screens it may have contributed to the translational gap. Understanding the complex genetic architecture of the locus, deciphering the functional impact of CHRFAM7A on α7 nAChR neurobiology and utilizing human-relevant models may offer novel approaches to explore α7 nAChR as a drug target.

## Introduction

Genes [1–4] that emerged since the human chimpanzee divergence have contributed to human-specific traits enriched for brain, immune and metabolic processes [1–4]. Genetic underpinnings of the differences include gene family duplications, single gene modifications, structural variants, differences in gene transcription levels and alternative splicing. *CHRFAM7A* is a human restricted fusion gene, a result of multiple rearrangements on chromosome 15 during evolution, including duplication, deletion and inversion events [5]. The region has ongoing instability, and various neurodevelopmental phenotypes have been reported in the context of deletion and duplication syndromes [3]. *CHRFAM7A* has been implicated in late-onset neuropsychiatric disorders, including schizophrenia, bipolar disorder, dementia with Lewy bodies, Pick’s disease, and Alzheimer’s disease (AD) [5]; all are human-specific and affect association cortices and higher cognitive function.

## *CHRFAM7A*: genetic architecture

**Fig. 1:**
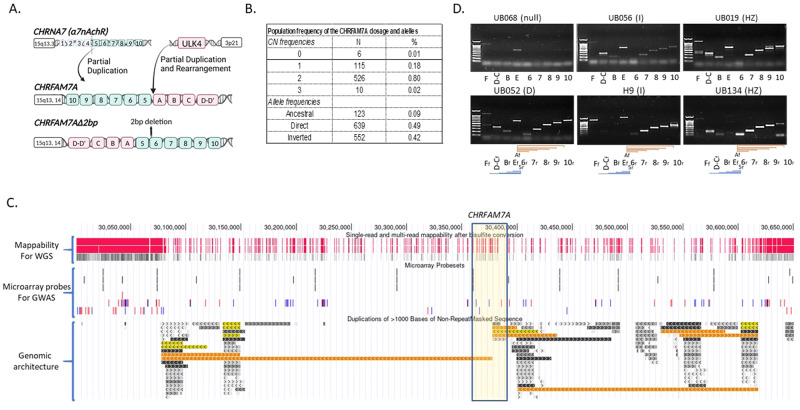
Genomic architecture of *CHRFAM7A* locus. **A** Schematic depicting *CHRFAM7A* alleles. **B** Copy number and allele frequency of *CHRFAM7A* in 657 normal controls by locus specific dual genotyping (TaqMan for dosage and Capillary sequencing for 2 bp deletion). **C** *CHRFAM7A* locus characteristics demonstrated in USCS genome browser reference sequence with active tracks including mappability which refers to the fidelity of sequence mapping to the reference genome, existing microarrays probes in frequently used commercial microarrays and the genomic architecture of low copy repeats depicted as orange and grey bars. These LCRs have over 95% sequence homology. These tracks indicate limited mappability (density of red bars); sparse probe coverage of *CHRFAM7A* in SNP arrays undermining detection of association in GWAS studies and complex genomic architecture with low copy repeats. **D** PCR mapping of the alleles in 6 samples with known *CHRFAM7A* genotype (depicted on top): UB068 – 0 copy of *CHRFAM7A*, null; H9 and UB056 - inverted, I; UB019 and UB134 - heterozygous, HZ; UB052 - direct, D. Primer sets are depicted below. To detect a part of *CHRNA7*, the forward primer was designed to hybridize with a unique sequence in exon A and the reverse primers - within exons 6, 7, 8, 9, and 10. To decipher the exon composition of *FAM7A/ULK4* segment, the forward primers are designed in sequences in exon F, D-C, B, E and the reverse primer - in exon 5 on *CHRNA7* segment.

*CHRFAM7A* is a fusion gene between part of *CHRNA7* (exons 5-10) and part of *FAM7A*. The unique sequence is limited to the breakpoint creating detection challenges (Fig. 1A). To add to the complexity, *CHRFAM7A* can be present in direct or inverted orientation. The inverted allele harbors a 2 bp deletion in exon 6 of the *CHRNA7* derived sequence leading to a frameshift mutation. Linkage disequilibrium between the 2 bp deletion and the inversion allows detection of the inverted allele.

Thus, unambiguous genotyping depends on two independent locus-specific assays: as a first step, TaqMan assay detects the dosage of *CHRFAM7A* alleles (possible copy number 0-3); as a second step, capillary sequencing detects the 2 bp deletion in exon 6. As exon 6 is present in *CHRNA7* (2 copies) and *CHRFAM7A* (0-3 copies), the ratio of the capillary sequencing peaks (2 bp deletion versus no deletion) deciphers the number of direct and inverted *CHRFAM7A* structural variants [6, 7]. *CHRFAM7A* allele frequencies by locus-specific dual genotyping have been studied in various cohorts [6, 8, 9]. The largest cohort of 1174 subjects of European descent revealed that *CHRFAM7A* is present in 0 (ancestral, 0.1%), 1 (18%), 2 (80%) and 3 (0.02%) copies in the human genome (Fig. 1B). Frequency of the direct allele is 49%, in contrast to 42% of the inverted and 9% of the ancestral allele [6]. Recently, inverted allele frequency was reported from the 1000 genome project revealing diversity in various populations, ranging from 8% in Africa to 66% in East Asia [10]. Of note, detection of the 2 bp deletion allele does not define the genotype, as it can represent hemizygous, heterozygous or homozygous state [10].

To add to the challenges, the genomic architecture is complex; *CHRFAM7A* and its parent sequences, *CHRNA7* and *FAM7A/ULK4* are embedded in a complex cluster of low copy repeats (LCR) (Fig. 1C). LCRs undermine genome assembly and lead to gaps and uncertainty in the reference genome, creating barriers to assay *CHRFAM7A* with genome-wide methods (Fig.1C). The region has limited mappability for single nucleotide polymorphism (SNP), copy number variation (CNV) array probes and next generation sequencing (NGS). All of these factors contribute to sparse SNP coverage and even less probe coverage for microarrays (Fig.1C). PCR mapping of 6 human donors demonstrates variance in the human population (Fig. 1D). In an NGS dataset, the breakpoint sequence is captured in 26% of samples in contrast to an expected frequency of 91% (data not shown).

This multiallelic genetic architecture embedded in LCR with the added complexity of the inversion positions *CHRFAM7A* in the blind-spot of genome-wide approaches. While algorithms to identify structural variants from whole genome sequencing (WGS) data have evolved over the years, these algorithms are limited to identifying relatively short inversions (<80kbp). These fundamental barriers for genotyping accuracy from whole genome approaches undermine the use of existing datasets. The elusive genotyping from microarray and NGS data underscore that *CHRFAM7A* has not been fully explored as a risk gene. Emerging long-range sequencing technology (TruSeq Synthetic Long Reads (TSLR), 10X Genomics linked- reads (10XG), Dovetail Genomics (Chicago Method), and Contiguity-Preserving Transposition Sequencing (CPT-Seq) [11] are promising alternatives; however, large datasets typically used in genome-wide association studies (GWAS) are not available. To be able to leverage existing microarray and WGS datasets, long-range sequencing of an adequate sample size of the human population is needed to fully understand diversity and the relationship of the *CHRFAM7A* alleles to ascertain genetic markers.

## Strong selective pressure

Genotype distribution of the 2 copy individuals is 25% direct homozygous, 25% inverted homozygous and 50% heterozygous, reminiscent of Hardy-Weinberg equilibrium. Based on the allele frequencies, the inverted allele underwent similar selective pressure as the direct allele, implicating that the inverted allele is functional. Both direct and inverted *CHRFAM7A* are transcribed as RNA in various cell types under normal and pathological conditions [12–14]. The direct allele is translated and modifies the α7 nAChR [6, 13, 15–17] which is a plausible foundation for driving selective pressure, although the mechanism is not known. The inverted allele is controversial; it is translated when overexpressed in SH-EP1 cells, oocytes, and Neuro2a cells-mouse neuroblasts [18, 19]. However, the expression vector was derived from the direct allele with in vitro mutagenesis deleting the 2 bp [18], thus interpretation of these results requires caution regarding translation of the nascent inverted allele. In silico analysis of the nascent inverted allele resulted in predictions including i) the protein is not translated as the distance between the Kozak consensus sequence from initiation makes native translation unlikely [20]; ii) or putative truncated peptide is translated due to the frameshift leading to an early stop codon; iii) or exons 5-10 from *CHRNA7* are translated with no unique sequence [14, 15]. More recent human iPSC functional readouts of the inverted allele indicate that the inverted allele is null, at least from the α7 nAChR perspective [6].

Further studies are needed to understand the function of the inverted allele and the underlying genetic mechanism. Small peptides that are not detectable by traditional assays [10, 18], and RNA-based mechanisms are emerging hypotheses in the human genome in general, with recent evidence from mining the “junk genome” [21, 22].

## Neuropsychiatric disease association

Despite the clear challenges with the locus, there is a signal that *CHRFAM7A* is a risk factor for neuropsychiatric diseases. Genetic association studies in neuropsychiatric diseases are summarized in Table 1.

**Table 1:** Genetic association and gene expression studies.

Disease	Gene	Design	Sample Size	Endophenotype	Cohort	Approach	Mutation	Effect	Odds Ratio	*P*-value	Ref
AD	CHRFAM7A	Pharmacogenetic	1174	Response to AChEI, DMT	TARCC	Candidate Gene	CN, 2 bp	Negative	NA	0.0048	[6]
AD	CHRFAM7A	Pharmacogenetic	63	Response to AChEI, acute	UB	Candidate Gene	CN, 2 bp	Negative	NA	0.037	[6]
AD	CHRFAM7A	Case/Control	1617	NA	ADNI	GWAS/Illumina Human610-Quad	CN	Negative	3.99; 95%, CI 1.49 –10.66	0.006	[23]
AD	CHRFAM7A	Case/Control	655	NA	ADNI, NIA-LOAD	GWAS/Illumina Human610-Quad	CN	Negative	NA	NA	[24]
AD	CHRFAM7A	Case/Control	781	Age at Onset	TARCC NIA-LOAD	GWAS/Affymetrix 6.0/Illumina Human610-Quad	CN	Positive	NA	0.018	[7]
AD	CHRFAM7A	Case/Control	175	NA	NA	Candidate Gene	2 bp	Negative	NA	0.011	[136]
Addiction	CHRFAM7A	Pharmacogenetic	408	Varenicline at 6 months abstinence	NA	Candidate Gene	CN	Negative	3.18, CI 1.09 –9.30	0.035	[29]
Bipolar disorder	CHRFAM7A	Familial, Case/Control	453	NA	NA	Candidate Gene	CN, 2 bp	NA	NA	NS	[8]
LBD	CHRFAM7A	Case/Control	35	NA	NA	Candidate Gene	2 bp	Positive	NA	0.001	[136]
Pick’s disease	CHRFAM7A	Case/Control	38	NA	NA	Candidate Gene	2 bp	Positive	NA	0.0001	[136]
Schizophrenia	CHRFAM7A	Familial, Case/Control	453	NA	NA	Candidate Gene	CN, 2 bp	NA	NA	NS	[8]
Schizophrenia	CHRFAM7A	Case/Control	251	Episodic memory	NA	Candidate Gene	2 bp	NA	NA	NS	[25]
Schizophrenia	CHRFAM7A	Case/Control	687	NA	NA	Candidate Gene	2 bp	Positive	OR 2.52 CI 1.31 –6.21, OR 1.49, CI 1.14 –2.42	0.015; 0.009	[27]
Schizophrenia	CHRFAM7A	Case/Control	871	Sensory gating	NA	Candidate Gene	2 bp	Positive	NA	0.004	[9]
Schizophrenia	CHRFAM7A	Case/Control	111/103	Antisaccadic movement	NA	Candidate Gene	CN, 2 bp	NA	NA	NS	[26]
Spinal cord injury	CHRFAM7A	Case/Control	45	Inflammatory markers	NA	Candidate Gene	2 bp	Positive	NA	0.015	[137]
Spinal cord injury	CHRFAM7A	Case/Control	27	Inflammation and neuropathic pain	NA	Candidate Gene	2 bp	Positive	NA	0.011	[138]
Addiction-Schizophrenia	CHRFAM7A	Post Mortem Brain	35	Gene expression	NA	Candidate Gene	Gene expression	Positive	Increased	0.009	[28]
Bipolar disorder	CHRFAM7A	Post Mortem Brain	35	Gene expression	NA	Candidate Gene	Gene expression	Positive	Increased	0.009	[28]
Bipolar disorder	CHRFAM7A	Post Mortem Brain	387 (700)	Gene expression	NA	Candidate Gene	Gene expression	Positive	Increased	0.01	[14]
Major Depression	CHRFAM7A	Post Mortem Brain	464 (700)	Gene expression	NA	Candidate Gene	Gene expression	NA	Increased	NS	[14]
Schizophrenia	CHRFAM7A	Post Mortem Brain	502 (700)	Gene expression	NA	Candidate Gene	Gene expression	Positive	Increased	0.0001	[14]
Anxiety	CHRNA7	Cases deletion	218	Behavioral, aggression	NA	Microarray	Structural variant	Positive	NA	0.032	[139]
Anxiety	CHRNA7	Familial	1 family	Behavioral, agression	NA	Microarray	Structural variant	NA	NA	NA	[140]
Anxiety	CHRNA7	Structural Variants	2886	Behavioral, agression	AGRE, BCH	Microarray	Structural variant	NA	NA	NA	[141]

*AD* Alzheimer’s Disease, *LBD* Lewy Body Dementia, *AChEI* acetylcholine esterase inhibitor, *DMT* disease-modifying treatment, *GWAS* genome-wide association study, *TARCC* Texas Alzheimer Research and Care Consortium, *UB* University at Buffalo, *ADNI* Alzheimer’s Disease Neuroimaging Initiative, *NIA-LOAD* National Institute of Aging-Late Onset AD, *AGRE* Autism Genetic Resource Exchange repository, *BCH* Boston Children’s Hospital.

AD and schizophrenia have been studied most, albeit in different ways. In AD, GWAS detected an inverse correlation of *CHRFAM7A* dosage with AD [7, 23, 24], without deciphering the direct and inverted alleles. In schizophrenia, a candidate gene approach was applied focusing on the 2 bp deletion; in addition, association with endophenotypes such as episodic memory and sensory gating were explored [8, 9, 25–27]. In schizophrenia, the presence of the inverted allele inferred the risk. *CHRFAM7A* expression was increased in post mortem brain tissue in schizophrenia, bipolar disease and major depression. The assay design does not distinguish whether the detected gene expression is from the direct or the inverted allele [14, 28]. The emerging pattern suggests that AD is associated with loss or reduced CHRFAM7A function, albeit agnostic to the orientation of the allele. In psychiatric diseases the inverted allele is associated with the disease state.

Pharmacogenetic studies in acetylcholine esterase inhibitor (AChEI) therapy in AD [6] and varenicline in addiction relapse [29] present the first proof of principle studies that *CHRFAM7A* is contributing to the translational gap in cholinergic therapies. With the understanding that 99.3% of humans harbor this human-restricted gene and that compound screens have a significant animal validation component without this human context, it is not surprising that treatment for AD and psychiatric disorders remains an unmet need.

## CHRFAM7A translated from the direct allele modifies the α7 nAChR

**Fig. 2:**
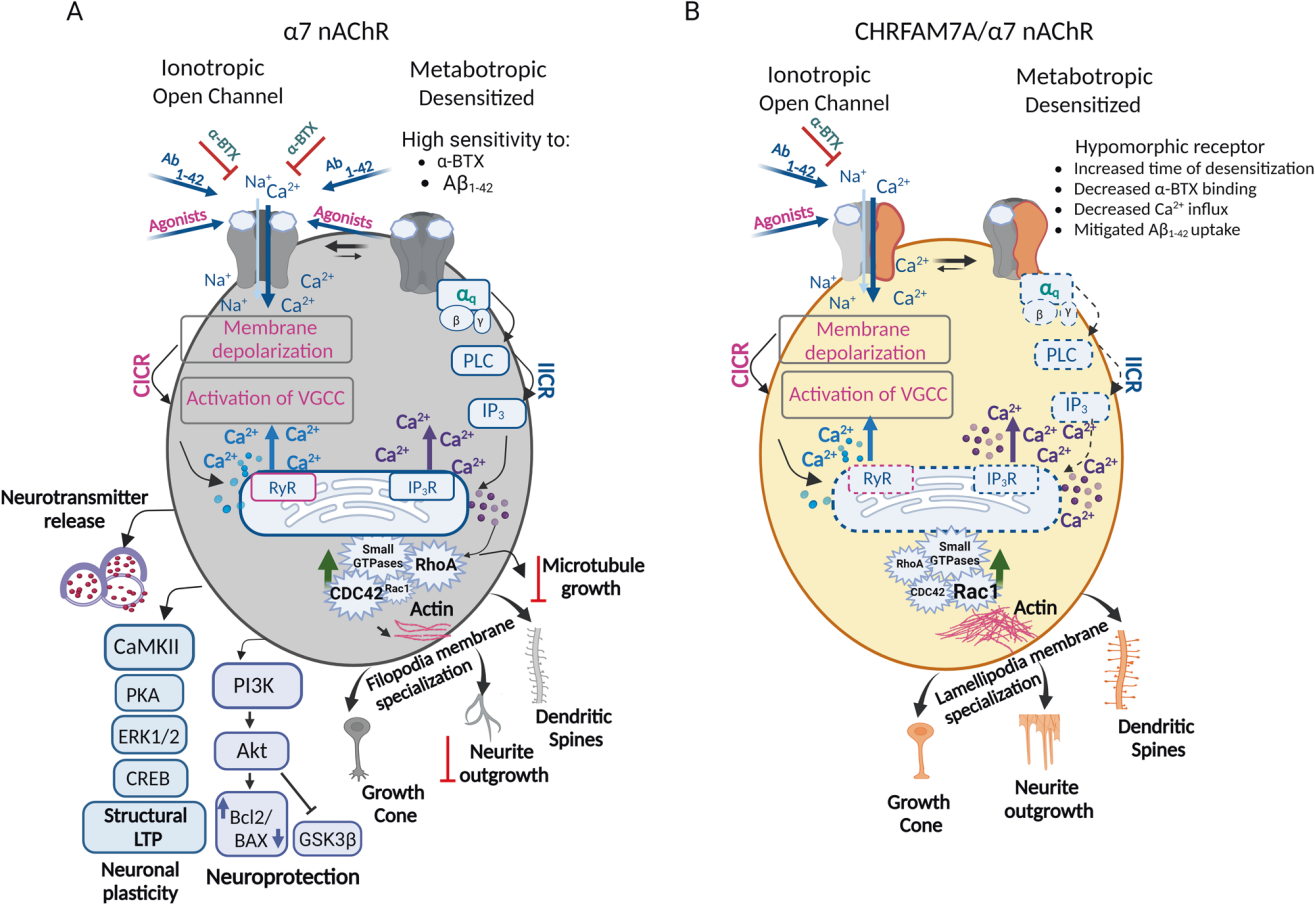
Gaps in understanding CHRFAM7A effect on α7 nAChR in the neuronal lineage. **A Schematic diagram illustrating α7 nAChR-mediated signaling cascades in neuronal cells**. Agonist binding to the α7 nAChR causes the receptor activation and an increase in Ca^2+^ concentration. Ionotropic receptor function is associated with Ca^2+^ influx from the extracellular space and calcium-induced calcium release (CICR) from the endoplasmic reticulum. The desensitized, inactive receptor is thought to function as a metabotropic receptor activating inositol 1,4,5-trisphosphate (IP_3_) induced calcium release (IICR) from the ER. Downstream Ca^2+^ signaling is implicated in 1) neurotransmitter release; 2) structural LTP (depends on sequential activation of Calcium–calmodulin (CaM)-dependent protein kinase II (CaMKII), protein kinase A (PKA), Extracellular signal-regulated kinase (ERK), and cyclic AMP response element binding protein, CREB; 3) activation of Phosphoinositide 3-kinase (PI3K) and Akt that leads to inactivation of glycogen synthase kinase 3 beta (GSK3β), and downregulation of apoptosis through downregulation of BAX and upregulation of Bcl2 that ultimately results in neuroprotection; 4) activation of RhoA that causes a decrease in actin and tubulin polymerization and attenuates neurite outgrowth and microtubule assembly; 5) activation of CDC42 that leads to filopodia membrane specialization in neurite outgrowth, growth cone, and dendritic spine. **B CHRFAM7A effect on α7 nAChR-mediated signaling pathways in neurons has been partially elucidated**. α7/CHRFAM7A nAChR being a hypomorphic receptor demonstrates decreased activation by electrophysiology and diminished Ca^2+^ influx. The hypomorphic receptor has decreased agonist (α-BTX) binding and mitigates amyloid beta 1-42 (Aβ_1-42_) uptake. α7/CHRFAM7A nAChR leads to decreased channel open probability shifting the time spent in CICR to IICR associated with activation of small GTPase Rac1. Downstream, Rac1 switches from CDC42/filopodia to Rac1/lamellipodia membrane structure at all levels of the neuronal unit: neurite outgrowth, growth cone, and dendritic spine. Compared to α7 nAChR (A), α7/CHRFAM7A nAChR associated phenotypes and signaling demonstrate significant gaps in knowledge (B) Dotted lines represent predicted pathways (created with BioRender.com).

α7 nAChR is a homopentamer receptor with a role in fast synaptic transmission and the highest Ca^2+^ conductor of the nAChRs (Fig. 2A). Translated CHRFAM7A retains the transmembrane and intracellular domains of CHRNA7, while the extracellular domain is truncated and consists of a unique protein sequence. CHRFAM7A can be incorporated into the α7 nAChR homopentamer as 1-3 copies, resulting in an α7/CHRFAM7A nAChR heteropentamer (Fig. 2B). In mammalian cells, CHRFAM7A subunits are assembled and transported to the cell membrane together with full-length α7 subunits [19, 30]. A physical interaction between CHRNA7 and CHRFAM7A was demonstrated using epitope- and fluorescent protein-tagged *CHRNA7* and *CHRFAM7A* constructs [19, 31].

The α7/CHRFAM7A nAChR functions as a hypomorphic receptor in various models utilizing transient or stable transfection [18, 19, 30, 32]. While there seems to be a consensus that CHRFAM7A alone cannot produce a functional receptor [18, 30, 32], data on the effect of CHRFAM7A on α7 nAChR specific currents are controversial [18, 30] likely due to differences in model systems and experimental designs. Electrophysiological studies on human iPSC-derived neurons indicate that CHRFAM7A affects PNU 120596-modulated currents of the α7 nAChR [6, 33]. While qualitatively the single-channel current clusters appeared similar in the presence or absence of CHRFAM7A, α7/CHRFAM7A nAChR demonstrated decreased channel open probability and faster run-down [6, 33]. Whole patch-clamp confirmed that the α7/CHRFAM7A nAChR is hypomorphic for the ionotropic effect [19].

Irreversible binding of fluorescent α-bungarotoxin (α-BTX), a selective α7 nAChR antagonist, has been utilized to investigate the effect of CHRFAM7A on α7 nAChR in vitro and in vivo. In rat neuronal PC12 cell line stably expressing CHRFAM7A, α-BTX binding was decreased [34]. α-BTX staining was diminished at the neuromuscular junction and brain tissue in CHRFAM7A transgenic mice compared to wild-type controls [34, 35]. Lower α-BTX binding was interpreted as less α7 nAChR expression on the membrane surface; an alternative interpretation of the data is that the assembled α7/CHRFAM7A nAChR is hypomorphic for α-BTX binding [18, 32]. Since in immune cells overexpression of CHRNA7 and CHRFAM7A results in α7/CHRFAM7A nAChR retention in the endoplasmic reticulum (ER) [31], both mechanisms may play a role depending on cell type.

α7 nAChR regulates intracellular Ca^2+^ pools [36]. In neurons, ligand-gated activation of α7 nAChR results in high Ca^2+^ permeability, followed by fast inactivation, extended desensitization and calcium-induced calcium release (CICR) (Fig. 2A). During the channel closed state α7 nAChR can function as a metabotropic receptor, albeit the metabotropic effect has not been fully elucidated due to challenges in the availability of controls [37]. The metabotropic effect is postulated to be through G-proteins (Fig. 2A), supported by structural analysis demonstrating that α7 nAChR possesses a G-protein binding cluster in the M3-M4 intracellular loop [38]. Coupling of α7 nAChR with the α subunit of heterotrimeric Gαq was demonstrated in neurons and with Gαi in microglia [39, 40]. Downstream, the signal transduction involves the activation of phospholipase C (PLC) and inositol triphosphate (IP_3_)- induced Ca^2+^ release (IICR) from the endoplasmic reticulum (ER) (Fig. 2A). Recently fluorescent live Ca^2+^ imaging revealed that CHRFAM7A reduces peak amplitude and area under the curve in single cell Ca^2+^ dynamics traces [41, 42]. The reduced Ca^2+^ signal is consistent with a hypomorphic α7/CHRFAM7A nAChR.

## CHRFAM7A in the central nervous system

A wealth of information is available on α7nAChR function from studies on cell lines (overwhelmingly from PC12 rat cells), rodent primary neuronal cultures and animal models [43]; in comparison data on how CHRFAM7A alters α7nAChR neurobiology is limited.

*CHRFAM7A* and *CHRNA7* are mostly co-expressed in the central nervous system (CNS) [14, 28, 44]. As both genes contribute to α7 nAChR in humans, CHRFAM7A functional readouts are expected where α7 nAChR is expressed, including neurons [45], astrocytes [46, 47], microglia [48, 49] and brain endothelial cells [50, 51]. Physiological implication of CHRFAM7A are not fully understood, but as a modifier of the α7nAChR it is plausible that CHRFAM7A plays a role in neuronal transmission [36, 52–54], neuroinflammation [55–57], neuroprotection [58, 59], vascular homeostasis [50] and the blood-brain barrier [50, 60]. In regards of cognitive domains, highest expression of α7nAChR is detected in the hippocampus, prefrontal cortex and amygdala [61–63]: areas involved in learning, memory, and attention [64–67]. How CHRFAM7A may affect these processes has recently gained some insights.

### CHRFAM7A function in neurons

In differentiated PC12 cells and animal models, α7 nAChR has been shown to contribute to fundamental neuronal processes (Fig. 2A). Presynaptically, α7 nAChR facilitates long-term potentiation (LTP) and inhibits long-term depression (LTD) [53]. Postsynaptically, it modifies downstream signaling leading to cyclic AMP response element binding protein (CREB) phosphorylation, changes in gene expression and modulation of neuronal activity (Fig. 2A). α7 nAChR is thought to regulate both the microtubule and actin cytoskeleton. In PC12 cells, α7 nAChR co-immunoprecipitates with Gαq and colocalizes with RhoA at the growth cone (GC); α7/ Gαq-dependent IP_3_ receptor (IP_3_R) phosphorylation, Phosphatidylinositol 4,5-bisphosphate (PIP_2_) breakdown, and α7/RhoA-dependent decrease in microtubule capping suggest that activation of α7-Gαq- IP_3_ pathway negatively affects microtubule dynamics [40, 68] (Fig.2A). α7 nAChR-G protein coupling performed in PC12 cells also leads to a RhoA associated decrease in actin polymerization and inhibition of neurite outgrowth [68]. In human iPSC-derived neurons, activation of α7 nAChR leads to increased CDC42 activity and filopodia membrane specialization at the growth cone and dendritic spine [42] (Fig. 2A). Actin polymerization and microtubule dynamics are the cytoskeletal foundation for neuronal plasticity.

Functional readouts on CHRFAM7A are emerging from *CHRFAM7A* transgenic mice and from post-mortem human brain (Fig. 2B). Proteomic profiling of *CHRFAM7A* transgenic mouse brain revealed that the presence of CHRFAM7A upregulates proteins involved in Ca^2+^ signaling, oxidative phosphorylation, as well as signaling pathways implicated in α7-nAChR-mediated neuropsychiatric disorders: AD, Parkinson’s disease, and Huntington disease [35]. Transcriptomic analysis of post-mortem human brain tissue identified Ca^2+^ signaling, small GTPases, synaptic structure and actin cytoskeleton being associated with increased *CHRFAM7A* expression [42].

Mechanistic insights into these omics-based hypotheses on CHRFAM7A-mediated neuronal phenotypes are studied in human isogenic iPSCs [42]. The model validated that CHRFAM7A modifies Ca^2+^ dynamics leading to the activation of small GTPase Rac1. Downstream, Rac1 creates a dynamic actin cytoskeleton and switches from CDC42/filopodia to Rac1/lamellipodia membrane structure at all levels of the neuronal unit, including neurite outgrowth, growth cone and dendritic spine [42] (Fig. 2B). Lamellipodia also facilitate adaptation to the mechanical properties of the tissue environment [42].

While CHRFAM7A is not a neurodevelopmental gene as null individuals are cognitively normal, brain vulnerability may be related to lower CHRFAM7A levels, explaining why AD is associated with lower CHRFAM7A dosage [7]. Human studies are needed to understand how this actin cytoskeleton gain of function (GOF) affects brain resilience, cognitive reserve and plasticity and to gain mechanistic insights into the neuropsychiatric disease associations.

α7 nAChR neurobiology exhibits other important mechanisms relevant to neurodegeneration. Amyloid beta (Aβ) binds the α7 nAChR with high affinity [69] and Aβ uptake induces neuronal toxicity [69] and apoptosis [70]. The hypomorphic α7/CHRFAM7A nAChR mitigates Aβ_1–42_ uptake and neurotoxicity in a dose-dependent manner [6, 33]. Intriguingly, mitigated Aβ_1–42_ uptake is associated with caspase-independent activation of inflammatory cytokines (interleukin 1beta (IL1B) and tumor necrosis factor alpha (TNFA)); suggesting a neuronal cry for help mechanism [33]. Pharmacological treatment with acetylcholine esterase inhibitors, AChEIs (donepezil, rivastigmine) and encenicline, a selective α7 nAChR agonist, revealed decreased efficacy in the presence of CHRFAM7A consistent with the α7/CHRFAM7A nAChR being hypomorphic [6]. In agreement with the in vitro observations, benefit from AChEI therapy was diminished in CHRFAM7A carriers in a human double-blind pharmacogenetic clinical trial [6]. These data resolve the conundrum that CHRFAM7A is associated with reduced risk of AD due to the neuronal structure GOF while response to therapy is mitigated because the α7/CHRFAM7A nAChR is hypomorphic.

### Microglia

**Fig. 3:**
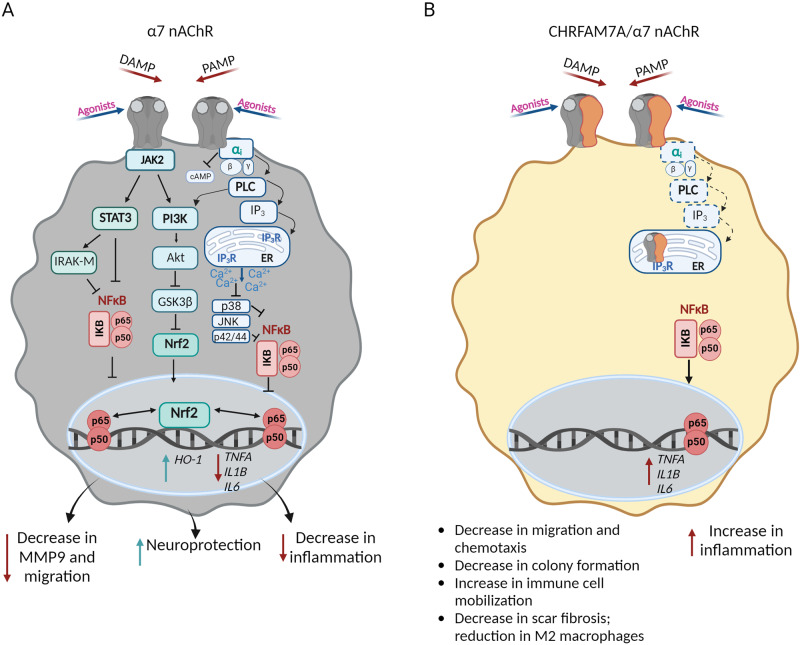
Gaps in understanding CHRFAM7A effect on α7 nAChR in the mononuclear cell lineage (microglia and macrophage). **A Schematic diagram illustrating α7 nAChR-mediated anti-inflammatory signaling cascades**. Activation of α7 nAChR leads to an inhibition of inflammatory cytokines by blocking nuclear factor -κB (NFκB) activity through: 1) Janus kinase 2 (JAK2)-signal transducer and activator-3 (STAT3); STAT3 activates interleukin-1 receptor-associated kinase M (IRAK-M). 2) Gαi-mediated pathway involving activation of phospholipase C (PLC), production of inositol 1,4,5-trisphosphate (IP_3_), its binding to the receptor (IP_3_R) in the endoplasmic reticulum (ER), which leads to Ca^2+^ release from the ER and causes deactivation of c-jun-N-terminal kinase (JNK), p38, and p44/42 mitogen-activated protein kinases. Activation of JAK2 also leads to activation of Phosphoinositide 3-kinase (PI3K) and Akt that phosphorylates and inactivates glycogen synthase kinase 3 beta (GSK3β), which, in turn, leads to activation and nuclear translocation of Nuclear factor erythroid 2-related factor 2 (Nrf2). Nrf2 induces the expression of anti-inflammatory heme oxygenase-1 (HO-1). Activation of α7 nAChR results in decreased inflammation, MMP9 expression and migration, leading to neuroprotection. **B CHRFAM7A effect on α7 nAChR-mediated anti-inflammatory signaling**. While signaling of the α7/CHRFAM7A nAChR in mononuclear cells are mostly unknown, emerging evidence suggests that the hypomorphic α7/CHRFAM7A nAChR releases NFκB inhibition leading to activation of proinflammatory cytokines (IL6, IL1β, TNFα). CHRFAM7A is associated with additional inflammatory phenotypes, including immune cell mobilization, a decrease in fibrosis and reduction in M2 macrophages and chemotaxis). Dotted lines represent predicted pathways (created with BioRender.com).

α7 nAChR is central to the cholinergic anti-inflammatory response by inhibiting nuclear factor kappa B (NFκB) activation and translocation to the nucleus [71], thus downregulates transcription of cytokines interleukin 6 (IL6), IL1B and TNFA [72–74]. In microglia, pharmacological modulation of the α7 nAChR with agonist PHA 568487 attenuated neuroinflammation and oxidative stress, while its antagonist methyllycaconitine, MLA, augmented them in the ischemic stroke model [75]. In α7 nAChR knockout (α 7nAChR^-/-^) mice ischemic stroke was associated with higher levels of proinflammatory cytokines (TNFα, IL6, IL-1β) [76]. α7 nAChR mediated signaling in these experiments involved inhibition of NFκB and an activation of nuclear factor erythroid-2-related factor 2 (Nrf2) leading to upregulation of antioxidant genes [71] (Fig. 3A). Although the signal transduction pathway has not been fully elucidated, there is evidence that α7 nAChR co-immunoprecipitates with Giα protein in microglia [39] consistent with the notion that α7 nAChR signaling in non-neuronal cells involves heterotrimeric G-proteins activation, IICR and effector kinases (Fig. 3A) [77].

In a hiPSC model of neuroinflammation, *CHRFAM7A* KI isogenic microglia demonstrated increased TNFα, IL6, IL-1β levels at baseline consistent with the release of the cholinergic anti-inflammatory tone due to the hypomorphic α7/CHRFAM7A nAChR [78] (Fig. 3B). When treated with lipopolysaccharide (LPS), a prototype pathogen-associated molecular pattern (PAMP), *CHRFAM7A* KI microglia demonstrated higher induction of IL-6 compared to null in a cytokine screen [78]. CHRFAM7A prolonged LPS induced NFκB nuclear presence and activation (Fig. 3B). Aβ_1–42_, a damage-associated molecular pattern (DAMP) that binds to α7 nAChR with high affinity, exhibited decreased Aβ_1–42_ uptake and increased innate immune response (decreased inhibition) in *CHRFAM7A* KI microglia [78]. While both the DAMP and PAMP responses are proinflammatory, Aβ_1–42_ has a direct effect on the α7/CHRFAM7A nAChR; in contrast, the LPS response is modulated by CHRFAM7A.

Similar to neurotoxicity, CHRFAM7A may be protective for microglia survival. In a cellular model of human cerebral ischemia-reperfusion (I/R) [79] overexpression of *CHRFAM7A* led to inhibition of microglia pyroptosis through NLRP3/ Caspase-1 and resulted in a diminished cerebral I/R injury [79]. In iPSC-derived microglia, the hypomorphic α7/CHRFAM7A nAChR mitigates Aβ_1–42_ uptake [78], which may serve as the mechanism for microglia protection.

Models are challenging due to the low abundance of the microglia relative to other cell types in the brain [80] and the limitations of immortalized microglia cell lines. Human iPSCs provide a theoretically unlimited resource, however, microglia differentiation protocols are burdensome and have low-yield [78, 81]. The field is evolving and alternative strategies, such as the use of monocytes and macrophages, are being explored [81, 82]. These efforts will facilitate deciphering the role of CHRFAM7A on microglia biology.

## CHRFAM7A in the immune system

The cholinergic anti-inflammatory pathway (CAIP) is a neuronal-immune interface, where the nervous system regulates immune function through a neurotransmitter [83]. Our understanding of the CAIP has significantly evolved [55, 84]; and the discovery of α7 nAChR expression in macrophages [57] was a critical step in this process. The neuronal-immune axis utilizes the efferent branches of the vagus and splenic nerves modulating α7 nAChR-expressing macrophages and T-cells [85]. The very nature of the interaction between two separate biological systems historically required in vivo studies in animal models and these experiments represent the foundation of our current understanding. Of note, animal models are agnostic to CHRFAM7A, thus our understanding is incomplete for 75% of the human population, those who carry the direct allele.

α7 nAChR is expressed in most cell types in the immune system [86], including both innate (macrophages, dendritic cells, basophils, and mast cells [87, 88]) and adaptive (T and B lymphocytes [89]) immune cells. In addition to its role in Ca^2+^ signaling (see microglia section), activation of α7 nAChR leads to signal transduction through Janus kinase 2 (JAK2)-signal transducer and activator-3 (STAT3) and/or Phosphoinositide 3-kinase (PI3K)/Akt signaling pathways [71, 90] (Fig. 3A). Phosphorylation of STAT3 negatively regulates NFκB, preventing its nuclear translocation and binding to DNA, thus decreasing transcriptional activation of proinflammatory cytokines [77] (Fig. 3A). Of note, attempts to target α7 nAChR in order to harness the anti-inflammatory mechanism also identified a translational gap in human clinical trials [91].

*CHRFAM7A* is abundant in human monocytes, macrophages, and monocytic cell lines [15–17, 92]. In macrophages *CHRNA7* and *CHRFAM7A* are independently regulated [92]. Treatment of THP-1 macrophage with LPS substantially decreased *CHRFAM7A* expression; this effect was prevented by an IκB kinase inhibitor, parthenolide, suggesting NFκB-dependent mechanism [15]. Thus, during inflammation, CHRFAM7A is downregulated which may serve as a negative loop to control the immune response by shifting abundance from α7/CHRFAM7A nAChR to α7 nAChR [15, 17].

CHRFAM7A-associated immune phenotypes were explored [20] using *CHRFAM7A* transduced cell lines [93] and a transgenic mouse model [13]. Consistent with the hypomorphic receptor, CHRFAM7A reduced cell migration, chemotaxis, and colony formation in THP-1 transduced macrophages [93]. CHRFAM7A KI mice demonstrated an increased hematopoietic stem cell reservoir in the bone marrow, myeloid differentiation, and a shift in cell population from granulocytes to inflammatory monocytes suggesting a role in hematopoiesis [13]. Consistent with the recently elucidated Rac1-actin reorganization [42], immune cells of CHRFAM7A KI mice demonstrated superior immune cell mobilization and invasion of the diseased lung and prevention of secondary bacterial infection [13].

CHRFAM7A is protective against inflammation-associated fibrosis. Overexpression of CHRFAM7A led to decreased fibrosis in the hypertrophic scar mouse model [94]. In this experimental paradigm, a reduction of M2 macrophages and activation of Notch signaling was observed in mice transfected with *CHRFAM7A*. In an obstructive nephropathy model overexpression of CHRFAM7A decreased the release of inflammatory cytokines in the kidneys and had a protective effect against renal fibrosis [95].

In human studies of sepsis and inflammatory bowel disease (IBD) *CHRFAM7A* expression is associated with augmented cytokine response consistent with the release of NFκB inhibition [12, 31, 96]. On the other hand, *CHRFAM7A* expression inversely correlates with HIV-associated neurocognitive disorders [44] and critical Covid-19 [97] suggesting a more efficient immune response. As inflammation plays a role in a broad spectrum of disease pathology, elucidating the full spectrum of the *CHRFAM7A* inflammatory phenotype may suggest novel treatment approaches [98].

## CHRFAM7A in cancer

Cigarette smoking is one of the most studied risk factors for cancer and has been associated with malignant neoplasms along the respiratory system (oral cavity, lung, pharyngeal) and remote sites [99, 100]. Nicotine, the prototypical nAChR agonist, and nicotine’s metabolic intermediates, nitrosamines 4-(methylnitrosamino)-1-(3-pyrydyl)-1-butanone (NNK) and N-nitrosonornicotine, emerged as the cause of this association [101]. α7 nAChR is expressed in several types of human cancer, including head and neck squamous cell carcinoma, bladder, squamous cell lung cancer cells, lung adenocarcinoma and small cell lung cancer [102, 103].

Features of the tumor environment, such as physical constraints, hypoxia, inflammation and metabolic stress activate complex signaling pathways known to play critical roles in both embryogenesis and tumor development. Experimental data on α7 nAChR from various cancer cell lines converge on phenotypes including cell proliferation, apoptosis, angiogenesis and metastatic potential; and inflammation in the tumor microenvironment [102].

**Fig. 4:**
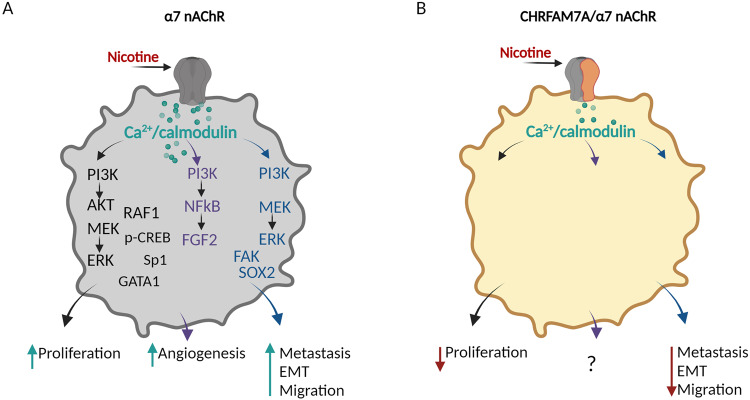
Role of α7 nAChR in cancer phenotypes. **A Schematic diagram illustrating α7 nAChR-mediated signaling pathways regulating cell proliferation, angiogenesis, and metastasis** (created with BioRender.com). Activation of α7 nAChR by nicotine leads to the activation of Ca^2+/^calmodulin-dependent signaling pathways increasing: (1) proliferation (Phosphoinositide 3-kinase (PI3K)/Akt; Mitogen-activated protein kinase/ERK kinase (MEK)/ Extracellular signal-regulated kinase (ERK); RAF1/Rb, and Sp1/GATA1); (2) angiogenesis (PI3K)/Akt/NFκB, FGF2); (3) metastasis, (4) epithelial-mesenchymal transition (EMT), and (5) migration (PI3K, MEK/ERK, focal adhesion kinase (FAK), and SOX2). **B CHRFAM7A effect on α7 nAChR-mediated signaling pathways in cancer**. Activation of α7/CHRFAM7A nAChR receptor by nicotine results in opposite phenotypes consistent with the hypomorphic response to an agonist: a decrease in proliferation, metastasis, EMT, and migration. Signal transduction is unknown (A) (created with BioRender.com).

There is a growing body of evidence that nicotine-mediated tumor progression is associated with the α7 nAChRs [104] (Fig. 4A). Although clinicopathological studies are sparse, increased α7 nAChR expression in cholangiosarcoma specimens is associated with higher histological grade (*p* < 0.01), tumor stage (*p* < 0.05), lymphatic (*p* < 0.01), and distant metastasis (*p* < 0.01). α7 nAChR expression also correlated with shorter survival (*p* < 0.0001) [105].

While CHRFAM7A in cancer has limited information (Fig. 4B), nicotine-associated tumor biology in the presence of CHRFAM7A is consistent with the hypomorphic receptor [106, 107]. In lung cancer, squamous cell carcinoma (SQC) specimens, the more nicotine-dependent type, had lower gene expression levels of *CHRFAM7A* in the peri-tumoral normal tissue compared to normal tissue in the less nicotine-dependent adenocarcinoma specimens (ADC). This suggests that in SQC the predominance of wild-type α7 nAChR may drive the nicotine association, while in ADC the hypomorphic α7/CHRFAM7A nAChR mitigates the role of nicotine. Of note, compared to normal tissue, CHRFAM7A was downregulated in both SQC and ADC [106] which suggests an interaction between CHRFAM7A and tumor biology.

The hypomorphic receptor decreases α7 nAChR mediated effects leading to decreased proliferation when exposed to nicotine, abrogated nicotine-induced epithelial-mesenchymal-transition (EMT), decreased migration and decreased nicotine-associated tumor growth of CHRFAM7A overexpressing xenografts [107]. However, invasion and inflammation, the two CHRFAM7A GOF phenotypes [42, 78] have not been studied in this report [107]. These emerging mechanistic insights into CHRFAM7A cell biology creates an opportunity to expand hypotheses regarding the role of CHRFAM7A in human cancer.

Beyond the mitigation of nicotine effect, CHRFAM7A has been shown to affect three fundamental biological processes that may be relevant to cancer: cellular response to the mechanical properties of the tissue microenvironment [42]; actin cytoskeletal changes leading to lamellipodia formation and invasion [42] and inflammation through NFκB activation, increasing IL-6 levels in particular [78]. All these pathways have been established in human cancer biology; the emerging experimental data positions CHRFAM7A as an upstream modulator of these processes.

### CHRFAM7A may play a role in tumor metastasis

Cellular motility is the foundation of the metastatic process, which includes invasion of tumor cells into the surrounding tissues and penetration of vessels and migration toward distant sites of the body away from the primary sites [108, 109]. Epithelial cancers lose cell polarity or undergo reprogramming leading to metastatic states dependent on microenvironmental signals and completion of the transdifferentiation process to promote cell motility. Actin restructuring drives the final outcome associated with invasion and migration phenotypes. During invasion cells rearrange their actin cytoskeleton, which creates special membrane structures called lamellipodia or invadopodia. In migration, cytoskeletal rearrangement results in cell elongation and directional motility [110]. The mode of motility is associated with distinct small GTPase activation [111, 112]

Human-restricted CHRFAM7A has been recently implicated in actin cytoskeleton dynamics and organization [42]. Mechanistically, the hypomorphic α7/CHRFAM7A nAChR leads to Ca^2+^ dynamic changes, resulting in Rac1 small GTPase activation [42]. Rac1 organizes the actin cytoskeleton to support lamellipodia and invasion [113]. In addition, the actin cytoskeletal reinforcement leads to adaptation to the mechanical properties of the tissue environments [42, 114, 115]; an important pillar of tumor biology [116]. As Rac1 is central to the metastatic process, CHRFAM7A likely has a significant role, especially in the invasion of the surrounding tissue and local spread for cancer. As Rac1 is an active drug target in metastatic cancer [117] understanding the role of CHRFAM7A has important translational significance [6].

### CHRFAM7A may alter the tumor environment through inflammation

As part of the complex process of metastatic behavior, tumor cells secrete small soluble proteins, such as cytokines, to stimulate neoplastic cells (autocrine effect) and prepare tumoral microenvironment (paracrine effect). Dysregulation of IL-6, a pleiotropic cytokine that plays an important role in multiple physiological processes, is associated with poor outcomes in cancer [118]. IL-6 pathway dysregulation contributes to cell proliferation through IL-6/JAK/STAT3 signaling and to cancer cell invasion [119–122]. CHRFAM7A has been shown to increase NFκB activation leading to cytokine release with highest effect on IL-6 in microglia [78]. An increased level of IL-6 was also detected in transgenic CHRFAM7A mice compared to wild type in an osteoarthritis animal model [123]. It is plausible that CHRFAM7A contributes to IL-6-associated cancer metastasis, implicating genotype-specific therapeutic potential.

Further studies are needed to understand the role of CHRFAM7A in tumor biology. Of importance, CHRFAM7A is not a cancer gene itself, rather gives a contextual human biology to cancer. As CHRFAM7A carrier status splits the human population 75% to 25% [6, 42], we expect profound translational significance for metastatic cancer treatment and immunotherapy.

## CHRFAM7A in other diseases

Although CHRFAM7A biology has not been studied in other organs and cell types, CHRFAM7A as a dominant negative modulator of the α7 nAChR is expected to modify α7 nAChR biology. Human data is available for α7 nAChR ionotropic effects on the cardiac conduction system from randomized clinical trials using α7 agonists and allosteric modulators. Of note, major effects on cardiac conduction has not been established [124, 125].

α7 nAChR effect on the heart is complex, involving both a direct ionotropic effect on heart rate and an immune-mediated effect connected to the vagus nerve and measured by heart rate variability. In clinical conditions associated with systemic inflammation (e.g. endotoxemia and sepsis), reduced heart rate variability and increased cardiac cycle regularity are observed. These effects can be triggered by LPS injection and IL-6 administration indicating a role of inflammation [126]. α7 nAChR is implicated in myocardial fibrosis, including right ventricular (RV) fibrosis, a maladaptive RV hypertrophy associated with poor outcomes in pulmonary hypertension [127].

In atherosclerosis, α7 nAChR mediates the immune response to cholesterol deposits, triggers the proliferation of vascular smooth muscle cells, and leads to oxidative stress and apoptosis [128–130]. In the pathomechanism of fatty liver and subsequent liver fibrosis, α7 nAChR was demonstrated to alter energy expenditure and O_2_ consumption, increase ECM expression and activate IL-6 [131]. In COPD, chronic bronchitis and lung fibrosis, α7 nAChR mediates increased mucin production and ECM expression [132, 133].

α7 nAChR attenuated experimental skin fibrosis in bleomycin-induced inflammation in mouse and human fibroblasts and the non-inflammation driven Transforming growth factor-beta (TGFβ) receptor I^act^ mouse model. α7 nAChR agonists reduced TGFβ1-mediated expression of collagen and myofibroblast. These actions were linked to modulation of the redox-sensitive transcription factor JunB and impairment of the mitochondrial respiratory system [134]. Anti-glomerular basement membrane antibody-induced glomerulonephritis in α7 nAChR KO mouse model exacerbated the glomerulosclerosis by increasing expression of fibrin, collagen TGFβ and TIMP-2 [135]. In CHRFAM7A transgenic mice with obstructive nephropathy, overexpression of CHRFAM7A decreased the release of inflammatory cytokines and had a protective effect against renal fibrosis [95]. Upregulation of *CHRFAM7A* gene expression and associated downregulation of *CHRNA7* expression was detected in patients with IBD [12].

α7 nAChR effect on several organs reveals a common theme of fibrosis and inflammation as a disease mechanism. As CHRFAM7A is a human-specific additional layer of immune regulation, further studies are needed in models incorporating CHRFAM7A to develop rational therapeutic approaches.

## Conclusion

CHRFAM7A remains an understudied area of neurobiology and for clear reasons. The genetic architecture is complex and the inversion event markedly diminishes the fidelity of the reference sequence and mappability, which resulted in the avoidance of this region in whole genome assay development. α7 nAChR biology accumulated substantial experimental data over the years on its role in brain function and disease, immunology and cancer. Clinical trials of drugs targeting the α7 nAChR have failed. Recent work on CHRFAM7A is starting to shed light on its biological function, suggesting that the biology of the hypomorphic α7/CHRFAM7A nAChR is distinctly different from the α7 nAChR readouts, which may contribute to the translational gap. Functional insights into the two alleles will inform genetic association and pharmacogenetic studies by refining the genetic model.

To fully understand the impact of CHRFAM7A in humans, there is a need to develop tools. Long-range sequencing is needed to build a reliable reference sequence of the locus and to understand human diversity. Human relevant models, that provide the human context have just started to emerge. The function of the inverted allele needs systematic exploration from human datasets. Once all of these are achieved, we can reiterate existing large datasets and re-analyze clinical trials with pharmacogenetic design. These are reasonable short-term goals which may lead to therapies for diseases where the unmet need is palpable.
